# Connaissance et volonté de prescrire la prophylaxie pré exposition (PrEP) par les prestataires des soins de santé à Kinshasa, République Démocratique du Congo (RDC)

**DOI:** 10.11604/pamj.2019.34.166.18025

**Published:** 2019-11-26

**Authors:** Benilde Izizag Bepouka, Hippolyte Situakibanza, Yamin Kokusa, Aliocha Nkodila, Francine Kizunga, Florian Kiazayawoko

**Affiliations:** 1 Service des Maladies Infectieuses et Tropicales, Département de Médecine Interne, Cliniques Universitaires, Faculté de Médecine, Université de Kinshasa, République Démocratique du Congo; 2 Clinique Marie Yvette, République Démocratique du Congo; 3 Hopital Général de référence de Kinshasa, République Démocratique du Congo

**Keywords:** Connaissance, volonté, prestataires de soins, PrEP, République Démocratique du Congo, Knowledge, willingness, care providers, PrEP, Democratic Republic of Congo

## Abstract

**Introduction:**

la réduction de l'incidence de nouvelles infections liées au VIH est un objectif de santé publique. L’objectif de l’étude était d’évaluer la connaissance et volonté de prescrire la PrEP à Kinshasa.

**Méthodes:**

il s’agit d’une étude transversale à visée analytique auprès des prestataires de soins de 4 structures de prise en charge de VIH/SIDA de la ville de Kinshasa d’avril à octobre 2017. Les analyses univariées et multivariées par régression logistique ont été effectuées pour identifier les facteurs associés à la connaissance et la volonté de prescrire la PrEP.

**Résultats:**

quatre-vingt-cinq prestataires ont répondu à l’enquête. Moins du quart des prestataires connaissaient la PrEP avant l’enquête et la moitié avait la volonté de la prescrire. La barrière à cet acte évoquée était la résistance (83%). Les facteurs associés à la connaissance de la PrEP étaient la spécialité d’infectiologie et l’expertise en VIH. Les facteurs associés à la volonté de prescrire la PrEP étaient l’âge supérieur à 40 ans, la spécialité d’infectiologie et l’expertise en VIH.

**Conclusion:**

la connaissance de la PrEP à Kinshasa était faible et seule la moitié des prestataires était disposée à la prescrire. Etre médecin infectiologue et expert en VIH était associé à la connaissance et la volonté de prescrire. Les futurs programmes d'éducation devraient renforcer la connaissance sur la PrEP et aborder les préoccupations identifiées dont les barrières à la prescription.

## Abstract (en)

**Introduction:**

reducing the incidence of new HIV infections is a public health goal. The purpose of this study was to evaluate the knowledge and willingness to prescribe PrEP in Kinshasa.

**Methods:**

we conducted a cross-sectional analytical study among care providers of 4 health facilities where HIV treatment was offered in the city of Kinshasa from April to October 2017. Univariate and multivariate logistic regression analyses were carried out to identify the factors associated with knowledge and willingness to prescribe PrEP.

**Results:**

eighty-five care providers responded at the survey. Less than one quarter of care providers knew PrEP before the survey and half of them were willing to prescribe it. Reluctance was due to resistance (83%). Factors associated with the knowledge of PrEP were the specialty of infectious disease and the expertise in addressing HIV. Factors associated with the willingness to prescribe PrEP were an age greater than 40 years, the specialty of infectious disease and the expertise in addressing HIV.

**Conclusion:**

the knowledge of the PrEP in Kinshasa was low and only half of care providers were willing to prescribe it. Being an infectious diseases specialist and an expert in HIV was associated with the knowledge and the willingness to prescribe PrEP. Education programs should strengthen the knowledge on PrEP and address concerns leading to reluctance to prescribe it.

## Introduction

Le virus de l’immunodéficience humaine (VIH) continue à être une cause majeure de morbidité et de mortalité dans le monde entier. Globalement, environ 2.1 millions des personnes étaient nouvellement infectées par le VIH en 2015 [1]. Déterminer chaque année le nombre de nouvelles infections à VIH, prévenir la transmission restent un but majeur pour les soins cliniques et la recherche. Les programmes de réduction de risque de transmission sexuelle, l’utilisation des condoms, le dépistage et le traitement des infections sexuellement transmissible (IST) servent comme stratégies préventives lesquels ont un impact sur la transmission globale [2,3]. La PrEP est l’utilisation des traitements antirétroviraux dans le but d’empêcher la contamination du VIH auprès des personnes séronégatives à risque substantiel de contracter le VIH. Il s’agit notamment des couples serodiscordants et les populations clés. La prophylaxie préexposition doit être fournie à des personnes ayant un risque substantiel d’infection à VIH et celles qui ont des risques continus d’exposition au VIH [4-6]. En RDC, elle est proposée aux personnes suivantes: couples serodiscordants, professionnelles de sexe, hommes ayant des rapports sexuels avec les hommes (HSH), utilisateurs des drogues injectables (UDI) et transgenre [7]. Des nombreux essais cliniques ont démontré l’efficacité des antirétroviraux de la PrEP [8-12]. Les récentes données indiquent une réduction effective de l’acquisition de l’infection à VIH parmi les injecteurs des drogues en utilisant chaque jour TDF comme prévention [10]. Plusieurs études dans le monde ont évalué la fourniture des soins de santé et les perspectives des prestataires sur la PrEP [13-15], mais aucune étude n’a évalué la connaissance et la volonté de prescrire la PrEP en République Démocratique du Congo. L’objectif de la présente étude était d’évaluer la connaissance et volonté de prescrire la PrEP par les prestataires des soins de santé dans la ville de Kinshasa.

## Méthodes

### Nature de l'étude

Etude transversale à visée analytique.

### Période et cadre de l'étude

L'étude s'est déroulée du 1^er^ avril au 1^er^ octobre 2017. Elle a été menée auprès des prestataires des soins choisis dans quatre structures sanitaires qui ont un service de prise en charge des personnes vivant avec le VIH/SIDA sélectionnées selon le niveau de prise en charge: les Cliniques Universitaires de Kinshasa pour le niveau tertiaire, l'Hôpital Militaire Kokolo et le Centre Hospitalier Monkole pour le niveau secondaire et le Centre Lisungi pour le niveau primaire.

### Population d'étude

Il s'agissait des prestataires des soins (médecins et infirmiers).

### Critère d'inclusion

Nous avons inclus les prestataires de soins ayant donné le consentement.

### Critère de non inclusion

Les prestataires des soins d'un service non prescripteur d'ARV n'étaient pas inclus.

### Echantillonnage

L'échantillonnage utilisé dans cette étude était de convenance.

### Collecte des données

Nous avons débuté par une descente sur terrain pour la prise de contact avec les responsables des différentes structures. Les données de cette étude avaient été collectées sur base de l'interview faite aux prestataires disponibles, ayant accordé leur consentement et cela à l'aide d'un questionnaire standardisé et pré testé. Le questionnaire était systématiquement proposé à chaque prestataire vu. Le questionnaire utilisé s'intéressait aux caractéristiques générales, la volonté de prescrire la PrEp, la connaissance de la PrEP, la préoccupation de prescrire, barrière à prescrire la PrEp par les prestataires et les pratiques actuelles de la prévention contre le VIH.

### Analyses statistiques

Après collecte des données, nous avons saisi et encodé les données sur Excel. Toutes les analyses étaient réalisées à l'aide du logiciel SPSS version 2.1. Les données qualitatives étaient exprimées sous forme de fréquence relative en pourcent. La recherche des facteurs associés à la connaissance et la volonté de prescrire la PrEP était réalisée à l'aide des tests de régression logistique par analyse univariée puis multivariée. La valeur de p<0,05 était considérée comme seuil de significativité statistique.

### Considérations éthiques

Avant de commencer l'enquête, nous avons obtenu une autorisation administrative du Service des Maladies Infectieuses de la Faculté de Médecine. Les chefs de service des différentes structures ont été impliqués dans cette démarche. Lors des entrevues, les principes du respect de la personne et la confidentialité ont été garantis et observés. Le consentement éclairé pour chaque participant à l'étude a été signé avant chaque entrevue. Les participants ont été informés du caractère anonyme de l'enquête.

## Résultats

### Caractéristiques générales des prestataires des soins

La présente étude a inclus 85 prestataires. Il y avait 60% des hommes et 40% des femmes. Les médecins représentaient 89% des enquêtés dont 7,1% des spécialistes en maladies infectieuses. Les prestataires dont l’âge était = 40 ans prédominaient (57,6%) ainsi que le sexe masculin (60%) avec un sexe ratio H/F de 1,5. Il ressort également que les assistants juniors dans cette étude étaient majoritaires (31,8%). Les prestataires ayant également la pratique non académique prédominaient (69,4%) ainsi que ceux n’étaient pas experts (74,1%) (Tableau 1).

**Tableau 1:** Caractéristiques générales des prestataires de santé

Variables	Effectifs	Pourcentage
**Age**		
<40 ans	49	57,6
≥40 ans	36	42,4
**Sexe**		
Masculin	51	60,0
Féminin	34	40,0
**Spécialité**		
Assistant junior MI	27	31,8
Assistant Senior MI	22	25,9
Médecin généraliste	21	24,7
Infirmier	9	10,6
Infectiologue	6	7,1
**Pratique hospitalière**		
Académique	26	30,6
Non académique	59	69,4
**VIH expert**		
Oui	22	25,9
Non	63	74,1

### Connaissance et volonté de prescrire la prophylaxie pré exposition du VIH

Il ressort que très peu des prestataires (17,6%) connaissaient c’est quoi la PrEP et près de la moitié des prestataires des soins n’avaient pas la volonté (52,9%) de prescrire la PrEP.

### Intérêt sur la prophylaxie pré exposition du VIH

Plus de la moitié des prestataires des soins (55,3%) se sentaient concernés à prescrire la PrEP. Ils avaient l’intention de prescrire beaucoup plus aux personnes qui utilisent les drogues injectables (98,8%). Les barrières à cette stratégie évoquées étaient la résistance (83%), la toxicité (76,5%) et le coût (20%) (Tableau 2).

**Tableau 2:** Intérêt sur la prescription de la PrEP

Variables	Effectifs	Pourcentage
**Préoccupation à prescrire la PrEP**		
Je suis quelque peu concerné	33	38,8
Je suis concerné	47	55,3
Je suis très concerné	5	5,9
**Personne à prescrire la PrEP**		
HSH	80	94,1
Usage de drogue IV	84	98,8
Prostitué	47	55,3
Couples sérodiscordants	68	80,0
**Barriere à prescrire la PrEP**		
Résistance	71	83,5
Toxicité	65	76,5
Cout	17	20,0
NSP	14	16,5

### Pratiques actuelles de la prévention du VIH et prophylaxie post exposition

Tous les prestataires des soins (100%) connaissaient que les préservatifs et l’abstinence sont des pratiques pivot pour la prévention et 50 (58,8%) prestataires n’avaient jamais prescrit la prophylaxie post exposition alors que 35 (41,2%) prestataires avaient déjà prescrit la prophylaxie post exposition (Tableau 3).

**Tableau 3:** Pratiques actuelles de la prévention du VIH et post exposition

Variables	n=85	Pourcentage
**Pratique**		
Usage préservatif	85	100,0
Abstinence	85	100,0
Dépistage VIH	19	22,4
Dépistage IST	15	17,6
Conseil comportemental	77	90,6
**Prévention post exposition**		
Oui	35	41,2
Non	50	58,8

### Déterminants de la connaissance sur la PrEP

En analyse univariée, les déterminants significativement liés à la connaissance sur la PrEP étaient le statut d’infectiologue, d’expert en VIH et l’ancienneté de plus de 2 ans sur le VIH. En analyse multivariée, le statut d’infectiologue (ORa 3,64, IC95%: 1,67-6,62; p=0,017) et d’expert en VIH (ORa 2,88, IC95%: 1,13-5,75; p=0,002) étaient indépendamment associés à la connaissance de la PrEP dans la population d’étude (Tableau 4).

**Tableau 4:** Déterminants de la volonté à PrEP

	Analyses univariées		Analyses multivariées	
Variables	p	OR (IC95%)	p	ORa (IC95%)
**Age ≥40 ans**				
Non		1		1
Oui	0,001	5,17(1,99-8,39)	0,019	3,52(1,23-5,08)
**Infectiologue**				
Non		1		1
Oui	0,003	4,88(2,24-6,84)	0,026	3,30(1,77-5,20)
**VIH expert**				
Non		1		1
Oui	0,003	6,00(1,82-9,78)	0,029	4,71(2,20-6,99)
**Année dans VIH**				
**≥ 2 ans**				
Non		1		1
Oui	0,003	3,00(1,82-5,78)	0,443	1,38(0,26-2,76)
Constante	-	-	0,015	0,032

### Déterminants de la volonté à PrEP

L’âge de prestataires ≤40 ans, le statut d’infectiologue, d’expert en VIH et l’ancienneté >2 ans dans le VIH étaient les déterminants de la volonté prescrire la PrEP en analyse univariée. Après ajustement en multivariée, l’âge ≤40 ans (ORa 3,52, IC95%: 1,23-5,08; p=0,019), le statut d’infectiologue (ORa 3,30, IC95%: 1,77-5,20 ; p=0,026) et l’ancienneté >2 ans dans le VIH (ORa 4,71, IC95%: 2,20-6,99; p=0,029) étaient les déterminants indépendants associés à la volonté à PrEP (Tableau 5).

**Tableau 5:** Déterminants de la connaissance sur la PrEP

	Analyses univariées		Analyses multivariées	
Variables	p	OR (IC95%)	P	ORa (IC95%)
**Infectiologue**				
Non		1		1
Oui	0,002	4,50(2,65-6,39)	0,017	3,64(1,67-6,62)
**VIH expert**				
Non		1		1
Oui	0,002	4,58(2,99-8,75)	0,002	2,88(1,13-5,75)
**Année dans**				
**VIH ≥2 ans**				
Non		1		1
Oui	0,002	3,58(1,99-5,75)	0,600	1,09(0,13-2,75)
Constante	-	-	0,002	0,003

## Discussion

La présente étude a évalué la connaissance et de la volonté de prescrire la PrEP par les prestataires des soins dans les structures sanitaires prenant en charge les PVVIH de la ville de Kinshasa. En dépit de la faible information des prestataires ou de réglementation sur la PrEP en RDC au moment de cette étude, très peu des prestataires aucours de la présente étude connaissait la PrEP et la moitié des prestataires de soins avaient la volonté de prescrire la PrEP bien que non encore disponible. Il y a une faible connaissance de la PrEP (17,6%) par rapport aux résultats des études sud-américaine de Ross *et al.* (69%) [13] et Tang *et al.* (57,5%) [14] ainsi qu’à l’étude nord-américaine de Tellalian *et al.* (90%) [15]. Cet important écart serait dû au fait que les prestataires sont plus sensibilisés en Amérique et les populations clés sont bien identifiées et suivies dans ces milieux contrairement en Afrique subsaharienne.

Il ressort de cette étude que la moitié des prestataires de soins (52,9%) avaient la volonté de prescrire la PrEP bien que non encore disponible. Ces résultats étaient semblables aux résultats de Palummieri *et al.* [16] mais différents aux résultats trouvés par Ross *et al.* [13], qui ont trouvé une proportion élevée de 87% des prestataires qui avaient la volonté de prescrire. Cette faible volonté de prescrire par rapport à l’étude de Ross *et al.* peut s’expliquer par le fait que la majorité des prestataires des soins de santé dans notre étude n’est pas informée de la PrEP et par le manque de politique de vulgarisation de cette stratégie ainsi que la faible sensibilisation des prestataires des soins. L’enquête menée en Italie sur les connaissances et les attitudes des prestataires de soins sur la PrEP et sur leur volonté de prescrire la PrEP dans deux villes à forte prévalence du VIH a révélé que la plupart (53%) étaient d’avis que la PrEP était une approche efficace de prévention du VIH. Cependant un faible pourcentage de prestataires (17%) ont déclaré avoir déjà prescrit de la PrEP [16]. D’autres études ont associé de manière similaire le niveau de connaissances à la volonté de prescrire la PrEP, soulignant que la formation des prescripteurs de la PrEP peut être un élément clé de sa mise en œuvre future [17,18].

Près de la moitié des prestataires de santé (55,3%) se sentaient concernés de prescrire la PrEP et voudraient prescrire beaucoup plus aux personnes qui utilisent les drogues injectables (98,8%). Ce résultat n’est pas conforme à celui trouvé par Ross *et al.* [13] et Sharma *et al.* [19] qui dans leurs études, les enquêtés se sentaient concernés à prescrire la PrEP aux hommes ayant des rapports sexuels avec les hommes avec respectivement 87% et 71%. Cette différence serait expliquée par notre culture africaine qui voit la pratique des rapports sexuels entre hommes comme répugnante et non légale et les pratiquants de cette forme de sexualité ne se font pas remarquer.

Les préoccupations ou barrière concernant la prescription de la PrEP comprenaient plus le développement d’une résistance (84%). Ce qui concorde avec l’étude menée par Ross *et al.* [13] qui ont aussi trouvé comme barrière chez 91% des prestataires le développement de la résistance. Les principaux obstacles détectés coïncident qualitativement avec ceux cités par d’autres auteurs. Sharma *et al.* [19] et Janier Sànchez-Rubio Ferràndez *et al.* [18] ont trouvé comme principal obstacle à la prescription de la PrEP le coût (91,9%). Cet obstacle a été plus évoqué par le fait qu’au Canada et en Espagne ce programme est déjà vulgarisé. Par ailleurs, il a été considéré que les obstacles les plus importants étaient ceux concernant l’augmentation des IST (infections sexuellement transmissibles), le développement potentiel de résistances et le coût [20]. Le comportement sexuel à risque, représente le talon d’Achille des stratégies de prévention et l’une des principales préoccupations face à une utilisation potentiellement répandue de la PrEP [21].

La présente étude a trouvé que tous les prestataires (100%) des soins connaissaient comme moyen de prévention l’usage de préservatifs et l’abstinence. Tandis que Ross *et al.* ont trouvé comme moyen de prévention selon l’ordre de fréquence l’usage de préservatif (85%), dépistage de comportement sexuel à risque (84%), dépistage des infections sexuellement transmissibles (64%), test HIV régulier (32%) et l’abstinence (33%) [13]. Cette diversité des mesures de prévention a été rapporté par Ross *et al.* par rapport à notre étude car la prévention combinée n’a pas encore été bien vulgarisée dans nos milieux.

En analyse multivariée, les déterminants significativement liés à la connaissance sur la PrEP étaient le statut d’infectiologue et d’expert en VIH. Ce résultat est lié à plusieurs formations et renforcements de qualité en matière du VIH auxquelles les infectiologues et experts ont beneficié par rapport à d’autres prestataires. Dans la présente étude en analyse multivariée, l’âge, le statut d’infectiologue et l’ancienneté >2 ans dans le VIH étaient les déterminants indépendants associés à la volonté à PrEP. L’étude de Ross *et al.* [13] a montré que l’âge jeune, le fait d’être un médecin stagiaire et d’être infectiologue étaient des prédicteurs significatifs de la volonté de prescrire la PrEP.

La différence d’âge entre les deux études serait due au fait que les prestataires avec assez d’expérience et un nombre élevé d’exercice de la médecine qui s’intéresse du domaine du VIH dans notre pays par rapport aux jeunes débutants. D’autre part nos populations d’étude différentes expliquent cet écart, nous avons travaillé sur une population incluant tous les prestataires œuvrant dans le secteur du VIH y compris les infirmiers et nous avons travaillé dans quatre institutions alors que Ross *et al.* [13] dans leur étude au Guatemala n’avait inclus que les médecins d’un seul hôpital.

### Limites de l’étude

La présente étude présente quelques limites. Premièrement, l’échantillonnage n’étant pas aléatoire influence la représentativité des participants. Deuxièmement, la taille de l’échantillon étant petite par rapport au nombre des prestataires et à l’étendue de notre pays.

## Conclusion

Moins d’un quart des prestataires connaissait la PrEP. La moitié des prestataires était favorable à la prescription de la PrEP. Les infectiologues et les experts dans le domaine du VIH étaient les déterminants de la connaissance et volonté de prescrire la PrEP. Actuellement, la pratique de la PrEP est très peu utilisée dans notre pays. Nous recommandons une sensibilisation des prestataires des soins sur la stratégie en vue d’une véritable implémentation.

### Etat des connaissances actuelles sur le sujet

- La PrEP est une méthode biologique de prévention efficace pour les populations clés;
- La PrEP s’utilise en combinaison avec les moyens traditionnels de préventions tels que l’usage des préservatifs et abstinence;
- La PrEP est une stratégie nécessitant une adhérence au traitement.

### Contribution de notre étude à la connaissance

- Faible connaissance de la PrEP par les prestataires de soins en RDC;
- Volonté de prescrire la PrEP par les prestataires encore faible en RDC;
- Les populations clés telles que les HSH et les transgenres qui devront bénéficier de la PrEP ne sont pas encore acceptés dans nos milieux.

## Conflits d’intérêts

Les auteurs ne déclarent aucun conflit d'intérêts.

## Contributions des auteurs

Tous les auteurs ont lu et approuvé la version finale du manuscrit.
